# A staged screening of registered drugs highlights remyelinating drug candidates for clinical trials

**DOI:** 10.1038/srep45780

**Published:** 2017-04-07

**Authors:** C. Eleuteri, S. Olla, C. Veroni, R. Umeton, R. Mechelli, S. Romano, MC. Buscarinu, F. Ferrari, G. Calò, G. Ristori, M. Salvetti, C. Agresti

**Affiliations:** 1 Department of Neuroscience, Istituto Superiore di Sanità, 00161 Rome, Italy; 2 Istituto di Ricerca Genetica e Biomedica, Consiglio Nazionale delle Ricerche, Monserrato 09042, Italy; 3 Center for Experimental Neurological Therapies, Sant’Andrea Hospital, Department of Neurosciences, Mental Health and Sensory Organs (NESMOS), Faculty of Medicine and Psychology, Sapienza University of Rome, Italy; 4 Department of Medical Science, Section of Pharmacology and National Institute of Neuroscience, University of Ferrara, 44121 Ferrara, Italy; 5 IRCCS Istituto Neurologico Mediterraneo (INM) Neuromed, 86077 Pozzilli, IS, Italy

## Abstract

There is no treatment for the myelin loss in multiple sclerosis, ultimately resulting in the axonal degeneration that leads to the progressive phase of the disease. We established a multi-tiered platform for the sequential screening of drugs that could be repurposed as remyelinating agents. We screened a library of 2,000 compounds (mainly Food and Drug Administration (FDA)-approved compounds and natural products) for cellular metabolic activity on mouse oligodendrocyte precursors (OPC), identifying 42 molecules with significant stimulating effects. We then characterized the effects of these compounds on OPC proliferation and differentiation in mouse glial cultures, and on myelination and remyelination in organotypic cultures. Three molecules, edaravone, 5-methyl-7-methoxyisoflavone and lovastatin, gave positive results in all screening tiers. We validated the results by retesting independent stocks of the compounds, analyzing their purity, and performing dose-response curves. To identify the chemical features that may be modified to enhance the compounds’ activity, we tested chemical analogs and identified, for edaravone, the functional groups that may be essential for its activity. Among the selected remyelinating candidates, edaravone appears to be of strong interest, also considering that this drug has been approved as a neuroprotective agent for acute ischemic stroke and amyotrophic lateral sclerosis in Japan.

The progress in the development of immunomodulatory therapies for relapsing-remitting multiple sclerosis could not be generalized to diseases where inflammation is not prominent, including the progressive forms of multiple sclerosis^1^,^2^. This reflects the complexities often seen by those attempting to develop effective therapies^3^ in disorders where a neurodegenerative component is present and the experimental models are not ideal. Moreover, in progressive multiple sclerosis, several mechanisms participate in the pathophysiology of the disease, affecting different cell lineages, often with asynchronous dynamics. It is therefore possible that single treatments will bring limited benefit^4^,^5^. In such a perspective that may include the need of combination therapies, it may be advantageous to count on a substantial number of drugs targeting different pathophysiological mechanisms^6^.

Drug repositioning is a consolidated strategy for tackling the above problems; it cuts the timeline and costs of drug development and reduces, to some extent, the need to rely on *in vivo* animal models, while preserving safety^7^,^8^,^9^. Moreover, thanks to repurposing efforts, academic research has been able to bring, with limited resources, an increasing number of compounds to phase 2 trials in multiple sclerosis and in rare neurodegenerative diseases^10^,^11^,^12^,^13^,^14^,^15^,^16^. Even in the case of failure to bring these drugs to licensing, these proof-of-concept studies are providing important contributions to our understanding of “druggable” pathophysiology in neurodegenerative diseases^17^.

Systematic approaches are being developed to fully exploit the potential of repurposing^18^,^19^, including phenotypic screenings which may have some advantages in diseases where the pathophysiology is poorly understood. This was in fact the approach taken by three recent studies that identified pharmacological agents that were able to stimulate the differentiation of oligodendrocyte progenitor cells (OPC) into myelinating oligodendrocytes^20^,^21^,^22^. However, because of the above mentioned multiplicity of damage mechanisms, and because of the characteristics of the identified agents – not always compatible with prolonged treatments in chronic diseases – there is still a strong need to identify drugs that may be better tolerated, and possibly combined in polytherapies. Finally, a much-needed accomplishment is the de-risking of investments on drug development processes, including investments on repurposed drugs. Hence, the opportunity to confront data from different drug screening efforts, possibly including studies with multi-tiered evaluation systems, will be important to reduce some of the factors that currently dissuade investment in these diseases.

Here, we report a new assessment of the myelin repair capability of registered drugs through multiple phenotypic screens. Our approach was characterized by: (*i*) a sequential screening method that enables statistical data integration and hit picking across multiple phenotypic screens, allowing a robust ranking and prioritization of the active compounds; (*ii*) the validation and analysis of hits and their analogs to identify their chemical functional features, an essential step for compound optimization.

## Results

### Identification of compounds that stimulate oligodendrocyte development in a multiple phenotypic screen

**Figure 1:**
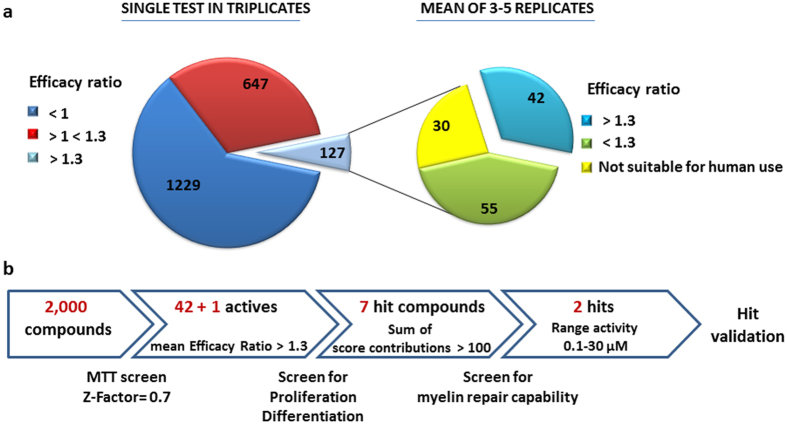
Cell-based screen identifies molecules with myelin repair potential. **(a)** Distribution of compounds in primary screen sorted by MTT Efficacy Ratio (n = 2,000). **(b)** Stepwise strategy followed to select 2 remyelinating hits for validation studies.

We selected the 2,000 compounds of the Spectrum Collection library to test for the development of OPC into myelin forming oligodendrocytes. Since OPC development is a process that demands high-energy, compounds were first screened for cellular metabolic activity in mouse purified OPC cultures by the MTT assay, a rapid colorimetric test of cell reducing activity (Z-factor = 0.7). Differences in OPC ability to reduce the tetrazolium salt MTT may be due to an effect of compounds on viability, proliferation or differentiation, all of which are important components of the remyelination process. Each compound was screened once in triplicates and an MTT value was quantified by an Efficacy Ratio defined as: ER = absorbance of drug/absorbance of vehicle. Platelet derived growth factor (PDGF, 20 ng/ml) and tri-iodothyronin (T3, 30 ng/ml) and thyroxine (T4, 40 ng/ml), known inducers of OPC proliferation and differentiation, respectively, were used as positive controls (PDGF ER = 1.4 ± 0.08 and T3 + T4 ER = 1.3 ± 0.05; mean ± SEM of 5 experiments run in triplicates). On the basis of the effect of both positive controls, we defined a threshold of ER = 1.3, singling out 127 positive compounds that were further analyzed in 3–5 experiments to confirm their activity (Fig. 1a). These experiments allowed for the identification of 42 compounds presenting a mean ER value ≥ 1.3 and are suitable for human use in their present chemical form (Table 1). This set included known classes of pharmaceutical drugs such as anti-inflammatory agents, vasodilators, antibacterials, antihypertensive agents, steroids, natural products as flavonoids, and others. Among these, 5-methyl-7-methoxyisoflavone which was incorrectly stated as methoxyvone in the Spectrum Collection library (see “Quality control” paragraph in validation section). Lovastatin, a drug that presented a mean value of ER = 1.23 ± 0.1 but with a demonstrated activity on OPC differentiation and myelin formation^23^,^24^ was added to this list to provide a convenient internal control for the screening. Moreover, statins are in an advanced phase of clinical evaluation in the therapy of multiple sclerosis alone or in combination with other drugs (www.clinicaltrials.gov).

We then moved to test the direct effect of the 43 (42 + lovastatin) selected compounds on the proliferation and differentiation of mouse purified OPC using specific assays (screen step in Fig. 1b). By [3 H]thymidine incorporation experiments, we established that about half of the compounds significantly stimulated the proliferation of OPC in different experiments compared to vehicle alone, with an increase that ranged from 1.5 to 7 fold compared to untreated cultures (Table 2, Prolif raw data ± SE). In parallel experiments, real time RT-PCR was used to assess the effect of the compounds on the expression of transcripts for ceramide galactosyltransferase (CGT), the key enzyme in the biosynthesis of myelin cerebrosides, and the myelin basic protein (MBP), a major component of the myelin sheath. We focused our analysis on CGT and MBP since they are markers of early and late stage of oligodendrocyte differentiation, respectively. Several compounds induced both CGT and MBP transcripts compared to vehicle alone, ranging from a 1.5 to 23 fold increase (median 4.1), and from 2.4 to 60 fold (median 5.6), respectively (Table 2, CGT and MBP raw data ± SE).

**Figure 2:**
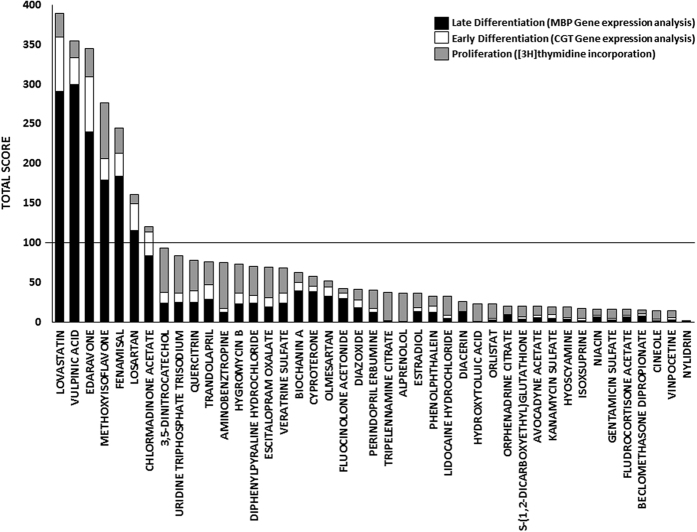
Compound effect on OPC proliferation and differentiation. Ranking of the best 43 hits according to the experimental data shown in Table 2. The scored contribution of OPC proliferation ([3 H]thymidine incorporation assay- grey), early differentiation (CGT Gene expression analysis- white) and late differentiation (MBP Gene expression analysis- black) experiments have been summed per-compound and all compounds have been compared according to their total and relative score. The seven drugs exhibiting an effect greater than 100 were selected for the next experimental setting. 5-methyl-7-methoxyisoflavone is abbreviated as methoxyisoflavone.

To select the best hits among the compounds active on the different biological processes analyzed, we ranked the substances by assigning a score to each experimental setting (see score assignment in methods section for details). Note that we rewarded differentiation more than proliferation as remyelination frequently fails due to a declining efficiency in the later stages of OPC development^25^. The score contribution of each compound was derived by multiplying the assigned score to the raw data of the experimental setting it belongs to (Table 2). Finally, score contributions have been summed per-compound and all compounds have been compared according to their total and relative score (Fig. 2). Seven compounds appeared to emerge from the others. We therefore took them as potential hit compounds. All the selected hits share remarkable properties such as neuroprotective, anti-inflammatory and anti-oxidant activities.

### Confirmation of hit compound myelin repair capability

To evaluate the remyelinating potential of the seven hits, we analyzed their activity in three biologically relevant systems of increasing complexity, sequentially discarding the compounds that were not able to achieve a significant effect in any one of the different *in vitro/ex vivo* tests (screen step in Fig. 1b).

Cultures of mixed glial cells containing astrocytes, oligodendrocyte lineage cells, and microglia were used to investigate compound effects on OPC developmental processes in the presence of mitogenic and differentiation factors secreted by the other glial cell populations. Cultures were incubated with each one of the seven compounds for 48 h, using T3 and T4 (30 and 40 ng/ml, respectively) as positive control (Table 3). Real time RT-PCR was used to analyze mRNA levels of CGT and MBP in three different experiments, showing that five compounds significantly up-regulated both transcripts (Table 3). The effect of the anti-inflammatory vulpinic acid and the anti-bacterial fenamisal did not reach statistical significance.

**Figure 3:**
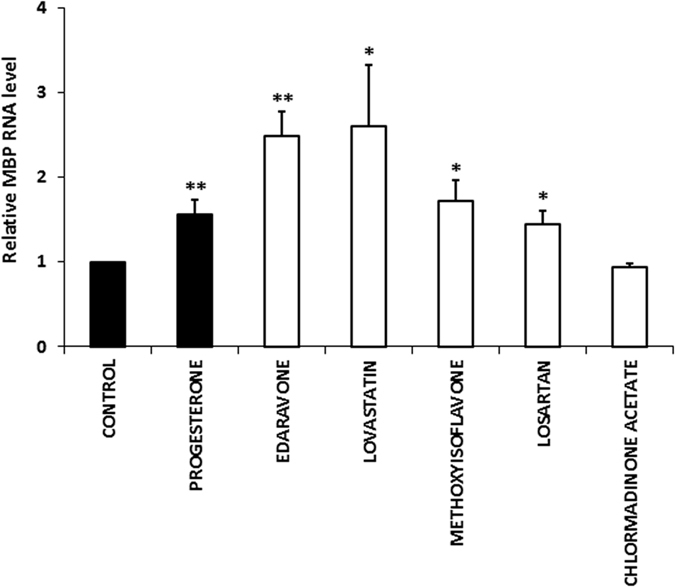
Compound induced MBP mRNA expression in cerebellar slices. Slices were prepared from P7 mouse cerebellum. Two hours after plating, slices were treated with compounds (20 μM) or DMSO (0.002% vehicle) for 5 DIV. Expression of MBP transcript was evaluated by real time RT-PCR. The results show that four compounds significantly (p* ≤ 0.05; p** ≤ 0.01) stimulated the expression of MBP mRNA compared to untreated control, demonstrating their ability to promote OPC differentiation. Data are expressed as 2^−ΔΔCt^ value relative to untreated control, using GAPDH as reference gene. Progesterone (40 μM) was used as positive control. 5-methyl-7-methoxyisoflavone is abbreviated as methoxyisoflavone. Bars represent the mean ± SEM of 3–5 independent experiments. (Student’s T-Test; *p ≤ 0.05; p** ≤ 0.01).

We next tested whether the five hit compounds could promote the differentiation of endogenous OPC in central nervous system (CNS) tissue. Cerebellar slices were generated from mice at postnatal day 7, a time that corresponds to the onset of myelination. Two hours after plating, slices were treated with compounds for 5 DIV and the expression MBP mRNA was evaluated by real time RT-PCR in 3–5 experiments. We focused on MBP transcripts since this myelin marker shows a wider range of expression than CGT during the developmental period analyzed. Progesterone (40 μM) was used as a positive control due to its myelin stimulating activity in this *ex vivo* model^26^. We found that only 5-methyl-7-methoxyisoflavone, edaravone, losartan and lovastatin significantly (p ≤ 0.05) increased MBP transcript levels relative to control slices, demonstrating their ability to promote OPC differentiation also in this experimental model (Fig. 3).

**Figure 4:**
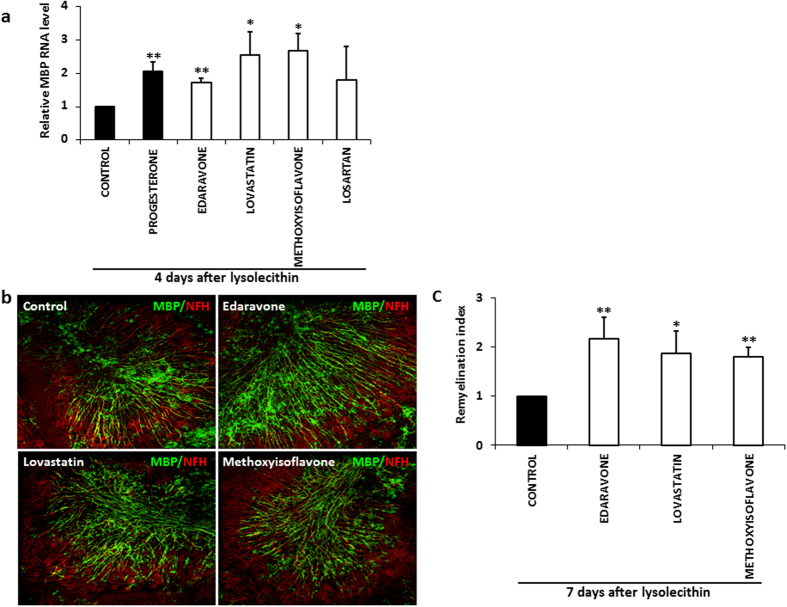
Compound induced MBP expression and remyelination in lysolecithin-demyelinated cerebellar slices. Slices were prepared from P10 mouse cerebellum, cultured for 7 DIV and then treated for 16 h with lysolecithin to induce demyelination. Immediately after toxin removal, cultures were incubated with compounds (20 μM) or DMSO (0.002% vehicle) for 4 (**a**) or 7 (**b** and **c**) days. **(a)** Total RNA was extracted and reverse-transcribed and MBP transcript expression was evaluated by real time RT-PCR. MBP mRNA fold increase was calculated as 2^−ΔΔCt^ value relative to untreated control, using GAPDH as reference gene. Results show that 3 compounds significantly enhanced MBP transcript compared to untreated control. Progesterone (40 μM) was used as positive control. **(b)** Immunostaining for MBP (green) and the axonal protein NFH (red). MBP staining increased and a major myelin alignment with axons was evident after treatment with the three analyzed compounds, relative to control. **(c)** Confocal quantification of remyelinated axons. The value of MBP/NFH co-localization was divided for the corresponding NFH staining to form the “remyelination index”. The rate of remyelination is significantly stimulated by all three compounds compared to control. Mean ± SEM of 3–5 independent experiments is shown. (Student’s T-Test; *p ≤ 0.05; p** ≤ 0.01)

Subsequently, we evaluated the remyelinating potential of the four selected compounds in toxin-treated cerebellar slice cultures, an *ex vivo* model of myelin damage where limited remyelination occurs in basal culture conditions. Cerebellar slices from P10 mice were cultured for 7 DIV and then treated for 16 h with lysolecithin to induce demyelination, as previously described^27^. Immediately after toxin removal, slices were incubated with the compounds (20 μM) or DMSO (0.002% vehicle) for the indicated times, using progesterone (40 μM) as positive control^28^. By real time RT-PCR, we demonstrated that after 4 days of incubation edaravone, 5-methyl-7-methoxyisoflavone and lovastatin, but not losartan, significantly (p ≤ 0.05) stimulated MBP transcripts compared to control slices (Fig. 4a). By a method of image confocal analysis quantification developed in house, we also evaluated the ability of the three hit compounds to induce an increase in axonal remyelination. In particular, the co-localization of the myelin protein MBP and the axonal protein NFH was analyzed in lysolecithin demyelinated slices maintained in the absence or presence of the compounds (20 μM) for 7 days. Consistent with the MBP gene expression analysis, we found that edaravone, 5-methyl-7-methoxyisoflavone, and lovastatin significantly increased MBP^+^ myelin membranes relatively to NFH^+^ axons compared to control slices (Fig. 4 b and c).

### Validation of edaravone and 5-methyl-7-methoxyisoflavone biological activity and chemical structure

By applying established procedures of the modern drug discovery pipelines, we validated the biological activity and chemical structure of the antioxidant edaravone and the flavonoid 5-methyl-7-methoxyisoflavone. The hypolipidemic compound lovastatin was not included in this analysis since, as previously mentioned, it was used as an internal control of the screening for its documented remyelinating activity^23^.

#### Quality Control

The analytical characterization of purity of the two hit compounds was performed by Proton Nuclear Magnetic Resonance Spectroscopy (1H-NMR) and Liquid chromatography–mass spectrometry (LC/MS) techniques. On the basis of the results obtained, we identified with certainty that the active compound of the screening is 5-methyl-7-methoxyisoflavone, which is incorrectly identified in the library data set as the flavone methoxyvone (7 methoxy-5methyl 2 phenylcromen 4-one) as the two compounds differ only for the position of the phenyl group (isoflavones and flavones present the C ring in position 2′ and 3′ of the B ring, respectively). Overall we confirmed that the chemical compounds analyzed correspond to edaravone and 5-methyl-7-methoxyisoflavone and present a purity of 97% and 99%, respectively (Supplementary Fig. S1), demonstrating that the activity depends exclusively on compounds and not on impurities (less than 5%).

#### Retesting

To confirm the compound activity and avoid false positives, we purchased independent stocks of the two hit compounds from different suppliers and tested their remyelinating potential in organotypic demyelinated slices. The increased level of MBP transcripts in slices incubated with the new stocks of edaravone and 5-methyl-7-methoxyisoflavone (20 μM, 4 days) compared to control slices confirmed the remyelinating potential of the two hits, demonstrating that the results obtained with the two compounds are not false positives (Supplementary Fig. S2).

#### Dose-response curves

**Figure 5:**
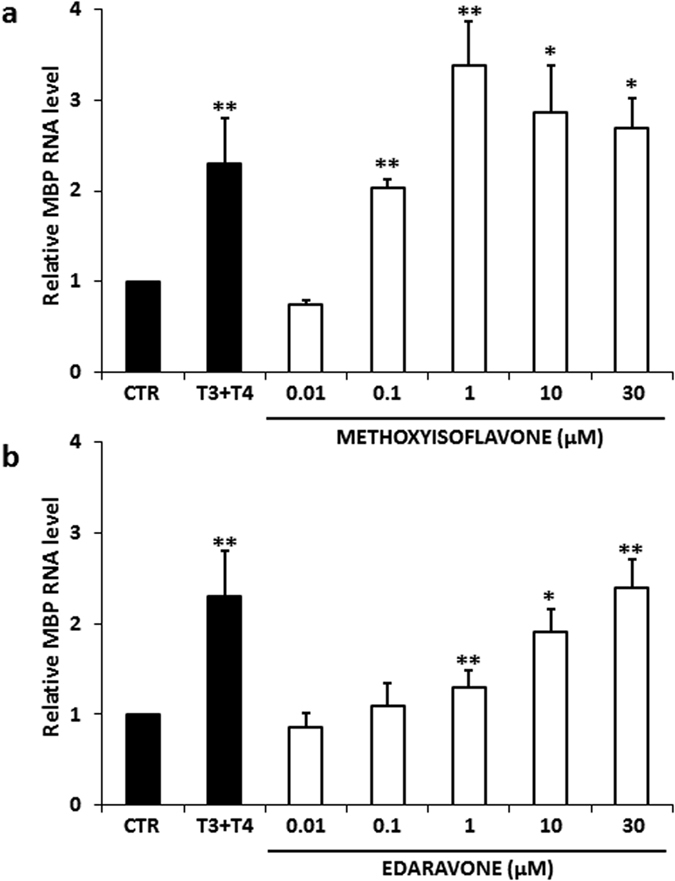
Dose-response curves of the two hit compounds in cultures of mixed glial cells. Cultures were prepared from neonatal mouse brain. After 9 DIV, the cells were incubated with **(a)** 5-methyl-7-methoxyisoflavone (methoxyisoflavone) and **(b)** edaravone at 5 different concentration (0.01–30 μM) for 48 h. Expression of MBP mRNA was evaluated by real time RT-PCR. The results shown that both compounds dose-dependently stimulated OPC differentiation. Data are expressed as 2^−ΔΔCt^ value relative to untreated control, using GAPDH as reference gene. Values represent the mean ± SEM of 3 independent experiments. (Student’s T-Test; *p ≤ 0.05; p** ≤ 0.01)

To assess the dose dependence of the assay’s readouts, we performed a dose-response analysis in cultures of mixed glial cells, as this is the most suitable test among those we had employed. Cells were incubated with edaravone and 5-methyl-7-methoxyisoflavone at five different concentrations (0.01–30 μM) for 48 h and expression of MBP mRNA was evaluated by real time RT-PCR. The results demonstrated that: i) the effect of both compounds was dose dependent and no activity was present at the dose of 0.01 μM, further confirming that there are no false positives; ii) the lowest and highest responses of 5-methyl-7-methoxyisoflavone were established at 0.1 and 1 μM concentration, respectively, with an evident decrease of the activity at 30 μM; whereas, edaravone showed a significant effect at the dose of 1 μM but it may not have reached its maximal efficacy at 30 μM (Fig. 5a and b). These results indicated that both compounds are active within a micromolar range but only edaravone did not show any toxic effect up to the concentration tested.

#### Chemical analog selection and testing

**Figure 6:**
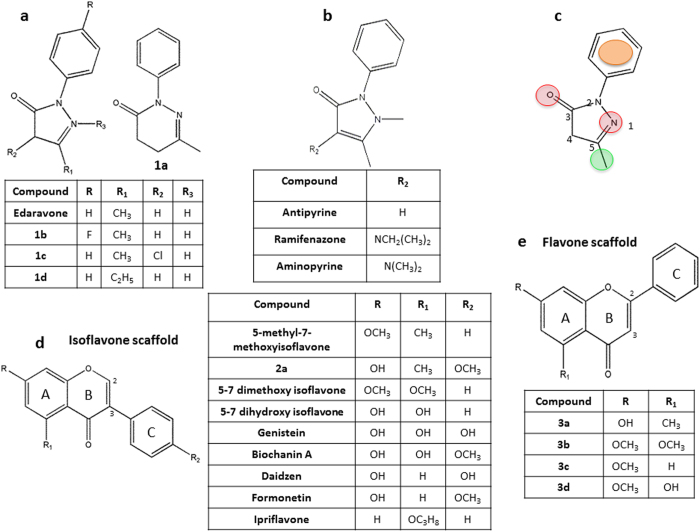
Activity of chemical analogs of edaravone and methoxyisoflavone and identification of edaravone chemical features. (**a**) The four edaravone analogs **1a–d** selected from commercial chemical libraries were used to confirm the structural class of edaravone structure and to identify its functional groups. Among these, only analogs **1b** and **1d** showed activity by MTT assay (see results). The activity of both analogs was confirmed in dose-response experiments and demyelinated cerebellar slices (Supplementary Figs S3 and S4). (**b**) The analogs antypyrine, aminopyrine and ramifenazone which were present within the Spectrum Collection library did not overcome the MTT test in both primary and re-testing experiments. **(c)** Basing on these findings four functional groups essential for the activity of edaravone were identified: in red 2 H bond acceptors (in position 1 and 3) plus in green hydrophobic group (the methyl in position 5) and in orange an aromatic ring (in position 2). (**d**) Nine isoflavones and **(e)** four flavones selected as analogs of 5-methyl-7-methoxyisoflavone were tested by MTT assay to confirm the compound activity. The analogs 2a and 3a–d were selected from commercial libraries, all the other compounds were present within the Spectrum Collection library. None of methoxyisoflavone analogs showed significant activity in both primary and re-testing experiments.

To identify a relationship between the structure and activity of two hits, we selected and tested structurally related chemical analogs. We selected new chemical analogs from commercial chemical libraries by means of a public database (Zinc, PubChem and ChemSpider), finding four available analogs for edaravone (Figs 1a–d and 6a,b) and five for 5-methyl-7-methoxyisoflavone, of which only one was an isoflavone and four were flavones (Figs 2a, 3a–d and 6d,e,). The activity of the selected analogs (10 μM, 48 h) was evaluated in OPC purified cultures by MTT test, identifying two active analogs for edaravone (**1b** ER = 1.36 ± 0.029; and **1d** ER = 1.44 ± 0.06) and none for 5-methyl-7-methoxyisoflavone. The biological activity of edaravone analogs was then confirmed in mixed glial cell cultures with dose-response curves at six different concentrations (0.001–30 μM; Supplementary Fig. S3) and in demyelinated cerebellar slices (Supplementary Fig. S4). By searching for chemical analogs in the Spectrum Collection library we found three analogs of edaravone (the antipyretic and analgesic drugs antipyrine, aminopyrine and ramifenazone; Fig. 6b) and 7 isoflavones (Fig. 6d) that did not overcome the MTT test in both primary and re-testing experiments. Since in the Spectrum Collection library many flavones and isoflavones are present, we selected only molecules with the same scaffold of the reference compound (Fig. 6d).

#### Chemical functional feature identification

By using the chemical structure of the two hit compounds as reference and their commercial and library analogs, we were able to identify some functional groups indispensable for their activity. As shown in Fig. 6c, we identified four functional groups essential for the activity of edaravone: 2 H bond acceptors (position 1 and 3, in red) plus hydrophobic group (the methyl group in position 5, in green) and an aromatic ring (position 2, in orange). These four groups are all present in active analogs: i) **1b** has a para-substitution on phenyl group in R, but the presence of a fluorine (a small and electronegative atom) did not prevent the activity; ii) **1d** is different from edaravone only for the presence of an ethyl group, which is always a hydrophobic group, in R1. These substitutions do not change the features responsible for the activity. On the contrary, the following substitutions result in loss of activity: i) the presence of an isopropylamino or dimethylamino or a chlorine group in R2, as in ramifenazone, aminopyrine and **1c**, respectively. Actually, the active compounds do not exhibit substituent in R2, suggesting that this position must remain empty; ii) the absence of H bond acceptor in position 1, as in antipyrine, ramifenazone and aminopyrine; iii) the replacement of the five-membered ring with one to six-membered, as in **1a**, which increases the distance between the hydrophobic group in position 5 and the H bond acceptor in position 3.

The lack of active analogs of 5-methyl-7-methoxyisoflavone among the few commercially available did not allow for the identification of functional groups essential for its activity. However, we were able to define that: i) the presence of a methoxy group in position R2 may prevent the activity, as in **2a** and in the library isoflavones biochanin A and formonetin (Fig. 6d); ii) the position of the ring in ’2 causes loss of activity; indeed, flavones **3a**, **3b**, **3c** and **3d** were inactive (Fig. 6e). Comparison between the chemical structure of 5-methyl-7-methoxyisoflavone and that of inactive isoflavones present in the library (Fig. 6d) showed that 5-methyl-7-methoxyisoflavone is the only compound that has a methyl group in R1, replacement with a methoxy group, as for 5–7 dimethoxyisoflavone (the two compounds differ only for this substituent), a hydroxyl group, as for 5–7 dihydroxyisoflavone, genistein, biochanin A, or a hydrogen, as for daidzein and formonetin, determines inactivity. These results suggest that the position in R1 must be occupied by a hydrophobic aliphatic substituent probably in combination with the absence of substitutions in R2. Finally, replacement in R with a hydroxyl group, as for genistein, daidzein, biochanin A or isopropoxy group, as for ipriflavone, are unfavorable for the activity.

### Prediction of compound targets

We used Chemical Similarity Network Analysis Pulldown (CSNAP) as the computational method to look for a possible consensus target that could explain the effect produced by the hit compounds edaravone, 5-methyl-7-methoxyisoflavone, and lovastatin. We included in the analysis other top scoring compounds of our screening (chlormadinone acetate, fenamisal, losartan, vulpinic acid) and four putative remyelinating compounds from other phenotypic screenings (benztropine, clemastine, clobetasol, miconazole; 20,21,22). Biochanin A, a compound that did not exceed the screening selection, was used as a negative control. CSNAP highlighted Neuropeptide S Receptor 1 (NPSR1; Uniprot Q6W5P4) and isocitrate dehydrogenase (Uniprot O75874) as possible consensus targets, showing the highest cumulative Schwikowski-score (S-score) across our hit compounds with a meaningful pattern across the other top scoring and putative remyelinating compounds (Supplementary Dataset S1) as well. The NPS G-coupled receptor was selected as a target for *in vitro* validation since, besides having the highest score, it belongs to a class of receptors that are implicated in many diseases and are the targets of numerous therapeutic drugs^29^.

The investigation of the pharmacological action of edaravone, 5-methyl 7-metoxyisoflavone, lovastatin, benztropine, losartan and biochanin A at recombinant NPSR was performed in HEK293 cells stably expressing the mouse NPSR by a calcium mobilization assay^30^. The results of these experiments showed that NPS increased the intracellular calcium concentrations in a concentration dependent manner with pEC_50_ and E_max_ values of 8.75 (8.30–9.20) and 335 ± 32% over the basal values, respectively. The compounds did not stimulate calcium mobilization up to a concentration of 10 μM (Supplementary Fig. S5). These compounds were then assayed as NPSR antagonists vs the stimulatory action elicited by NPS. In this series of experiments the selective NPSR antagonist SHA 68 was used as a standard. At 100 nM, SHA 68 was able to elicit a rightward displacement of the concentration response curve to NPS with a pA_2_ value of 7.83 (7.65–8.01). At the concentration of 10 μM, compounds did not affect the concentration response curve to NPS (Supplementary Fig. S6). Therefore, the tested compounds do not interact with the NPSR, either as agonists or as antagonists.

## Discussion

Multiple sclerosis pathophysiology is highly complex and difficult to reduce to *in vitro* or *in vivo* models where to investigate for new therapies. Nonetheless, high-throughput screenings for myelin repair are beginning to yield the first, consistent results^20^,^21^,^22^. However, because of the above complexity, false negatives and false positives are and will be inevitable. To limit them, comparisons of multiple screenings within and across studies are much needed. For the same reasons, and given the high numbers of compounds to be screened, it is important that these studies are structured as to balance inclusiveness and selectivity of the analysis.

CNS remyelination is the result of a specific sequence of events that are tightly regulated and recapitulate the process of oligodendrocyte generation that occurs during development^25^. Our staged series of assays, composed by multiple *in vitro* screens, allowed for the testing of compound effects on different aspects of OPC development and for the selection of the best hits by assessing their activity across the combined data set rather than their status within a single analysis. This multiple phenotypic screening highlighted a substantial number of compounds that are suitable for human use. These molecules belong to various classes of pharmaceutical drugs and represent useful information for further research in the field.

Three drugs, lovastatin, edaravone and 5-methyl-7-methoxyisoflavone, convincingly demonstrated activity in all the tiers of the screening process; their remyelinating potential arose from an increase in OPC proliferation and differentiation, which are essential components of the remyelination process. Based on the evidence that remyelination failure is more commonly associated with the impairment of the final stage of oligodendrocyte developmental program^25^, strategies to enhance remyelination are frequently designed to promote OPC differentiation into new myelinating oligodendrocytes^31^. However, several remyelinating molecules have been described to promote both proliferation and differentiation^32^,^33^,^34^ or proliferation alone^35^. These two processes are not mutually exclusive for the progression of oligodendrocyte lineage; indeed cAMP response element-binding protein (CREB), implicated in controlling myelin gene expression and oligodendrocyte generation, participates in the regulation of both OPC proliferation^36^ and differentiation^37^.

The biological plausibility of lovastatin as drugs with myelin repair potential confirms the validity of our approach and reinforces the rational for pursuing the clinical evaluation of lipophilic statins in the therapy of multiple sclerosis.

The hit compound edaravone is a free radical scavenger, approved as a neuroprotective agent for acute ischemic stroke in Japan, where it has been used for over 10 years. Surprisingly, it does not have market authorization in other countries^38^. It is a small bioactive molecule that easily crosses the blood brain barrier and has been indicated as a promising drug candidate for several neurodegenerative diseases^39^,^40^. Edaravone was also investigated in amyotrophic lateral sclerosis patients^41^ and recently received approval, again in Japan, for this disease. With respect to multiple sclerosis, encouraging studies in the model of experimental allergic encephalomyelitis already exist^42^. It is of interest that edaravone rescues oligodendrocyte differentiation after prolonged ischemic damage in mice^43^, reduces white matter injury in a mouse model of hypoxic-ischemia^44^, and has anti-inflammatory effects in activated microglia^45^. The identification of two active analogs of edaravone and the purity analysis validated its biological and structural activity, allowing us to conclude that the remyelinating activity of edaravone is not a false positive. The micromolar concentration range of activity and the lack of cytotoxicity shown by edaravone in our experimental models are consistent with previous studies performed on different cellular types, including all components of cerebrovascular units^46^,^47^. The weak activity of edaravone does not seem to be a major obstacle to its human use as the drug is registered not only for an acute disease (ischemic stroke), but also for a chronic condition (amyotrophic lateral sclerosis). Moreover, there are ongoing attempts to improve its poor oral bioavailability. If successful, these attempts may not only promote its use in chronic diseases but possibly also increase the commercial interest about its development as a licensed drug in different conditions^48^,^49^,^50^.

On these grounds, we suggest that it is urgent to initiate the clinical evaluation of edaravone in multiple sclerosis, considering also that based on our structure-activity relationship analysis, the molecule may be chemically modified to enhance its activity: four functional groups appear to be essential for the activity together with the presence of a five-membered ring that allows for the correct distance between the hydrophobic group in position 5 and the H bond acceptor in position 3.

The other hit of our screening, 5-methyl-7-methoxyisoflavone, is a semi-natural isoflavone. Isoflavones are the main group of flavonoids and are well characterized for their estrogenic and/or antioxidant properties. They are active ingredients of human diet since they are present in many daily-consumed foods, like vegetables and fruits. As plant-derived secondary metabolites, and in spite of some limitations about their final development as drugs^51^, they are relevant for drug discovery since they have a wide range of molecular targets. Their neuroprotective action may depend on estrogenic and other properties. 5-methyl-7-methoxyisoflavone has never been considered as a candidate for CNS repair. It shows some differences in chemical structure compared to traditional isoflavones: a methoxy group in R, found in some isoflavones, an unusual methyl group in R1 (Fig. 6d), but no hydroxyl groups, which are typical of flavonoids with mainly antioxidant activity. In addition, the lack of structure similarity to 17β-estradiol (the most potent estrogen in mammals) suggests that the activity of 5-methyl-7-methoxyisoflavone in our assays may not be related to the estrogenic properties typical of isoflavones such as genistein and daidzein, an interpretation that is supported - to some extent - by the inactivity of genistein and daidzein in our primary screening. Unfortunately, at variance with edaravone, the analogs of 5-methyl-7-methoxyisoflavone were not enough to allow the identification of a clear SAR. Driven synthetic modifications will be needed to improve pharmacological properties of this compound.

Overall, the possibility that the antioxidant action of the two hit compounds is not primarily responsible for their regenerative effect is supported by the fact that many other antioxidant compounds of the library did not show any activity in the screening process. Based on these considerations, the investigation of the molecular targets and pathways activated by edaravone and 5-methyl-7-methoxyisoflavone stands a good chance to uncover novel biological activities relevant to the regenerative process of remyelination. Apart from the elements of internal consistency of the data, and those in accord with published results from other groups, there are compounds that have demonstrated myelin repair potential in other experimental systems but whose efficacy did not emerge in our screening. In particular, the class of anticholinergic compounds with anti-muscarinic activity was not represented among our best hits in spite of the positive data from two different studies. While benztropine and clemastine had been identified as potential remyelinating compounds in screening assays performed at 1 μM final concentration^20^,^21^, they showed toxicity in our primary screening performed at 10 μM concentration. In MTT re-testing experiments, we confirmed the activity of both compounds at the lower concentration (1 μM, data not shown). This is consistent with the issue of low difference between active and toxic doses described for benztropine^20^.

The optimization of therapeutic algorithms, or the development of new molecules with better therapeutic indexes, would benefit from better knowledge about the targets that are relevant for the regenerative effects of these drugs. So far, and including this study, none of the screenings have unequivocally defined the therapeutic target(s) of the identified compounds. This is not uncommon (approximately, only the 20% of the small molecule drugs have an identified individual molecular target) and may reflect the need for a promiscuous mechanism of action for “neuroprotective” drugs to be effective. Interestingly, some of the recently identified drugs with myelin repair potential might share a common mechanisms of action; it has been recently shown that edaravone effects are mediated not only by the elimination of oxidative stress, but also by the increased production of brain-derived neurotrophic factor (BDNF) through phosphorylation of CREB^52^. Similarly, lipophilic statins may exert neurotrophic functions through the transcriptional activation of CREB and consequent BDNF increase^53^. Besides, the phosphorylation of CREB is also part of the cascade triggered during benztropine-induced oligodendrocyte differentiation^20^ and is involved in the regulation of OPC differentiation by GPR17, another potential target for the treatment of myelin damage^54^.

In conclusion, in spite of differences in the experimental platforms, and in the composition of the libraries of compounds, there are elements of consistency between the last screening studies on the remyelinating potential of small molecules. Moreover, all hits that emerged from the last four (20,21,22 and the present one) screenings are molecules that are registered for human use. This means that, while additional studies with different platforms are needed, there are already enough drugs to design experimental medicine trials to explore the clinical efficacy of the compounds (also in combination therapies) and increase our understanding of the biology of the disease. The results of this study and the clinical effects in two other neurological diseases strongly suggest that edaravone is one of the most interesting candidates. Future work will need to concentrate more on medicinal chemistry studies to optimize the compounds and increase the interest for industrial development. However, all these advancements also urge the support of political steps and regulatory solutions to facilitate the entire process of repurposing up to a full marketing authorization.

## Materials and Methods

### Experimental design

The goal of this study was to identify new therapeutic strategies for myelin repair that can be readily transferred to patients with demyelinating diseases of CNS. We carried out an unbiased *in vitro* and *ex vivo* screening in mammalian cells with the aim of repositioning drugs according to their remyelinating potential. The compound activity was first verified on the development of mouse OPC through multiple, sequential phenotypic screens and their myelin repair capability was then assessed in organotypic mouse brain slice cultures. The biological activity and chemical structure of hit compounds were validated, also allowing for the identification of their chemical functional features.

### Animals

CD1 Swiss mice were purchased from Harlan Laboratories (San Pietro Al Natisone, Udine, Italy). The experimental procedures related to the use of CD1 Swiss mice were performed in accordance with the approved national law with the implementation of European Union Directive nr. 86/609/CEE - D.lgs 116/92 regarding the Protection of animals used for experimental research. The experiments were approved by the Service for Biotechnology and Animal Welfare of the Istituto Superiore di Sanità and by the Italian Ministry of Health (Authorization #271/SSA/2010 -18/03/2010).

### Compound sources

The library tested was The Spectrum Collection (Micro- Source Discovery Inc., Groton, Conn.). An alphabetical list of the compounds is available at the Micro-Source Discovery website at www.msdiscovery.com/spectrum.html. The compounds are supplied as 10 mM solutions in dimethyl sulfoxide (DMSO). Edaravone and 5-methyl-7-methoxyisoflavone used in retesting experiments were purchased from Sigma-Aldrich and Ambinter (Orléans, France), respectively. Chemical analogs were purchased from Ambinter (2a and 3a), and from MolPort (Riga, Latvia; 1a–d and 3b,d).

### Staged screening of the compounds

#### Primary screen

We screened 2,000 compounds of The Spectrum Collection library comprising primarily Food and Drug Administration (FDA)-approved compounds or natural products. Purified OPC obtained from neonatal mouse primary mixed glial cultures (for details see Supplementary methods) were incubated with or without drugs at 10 μM concentration in dimethyl sulphoxide (DMSO; 0.001% vehicle) for 48 h. PDGF(20 ng/ml, Preprotech, Rocky Hill, NJ) and T3 (30 ng/ml; Sigma-Aldrich, St Louis, MO) and T4 (40 ng/ml; Sigma-Aldrich) were used as positive controls. The 10 μM concentration was selected to obtain more primary hits and more chemical scaffold types while simultaneously removing compounds that may have problems of toxicity^55^. Compounds were first screened for their ability to reduce [3-(4,5-dimethylthiazol-2-yl)-2,5-diphenyltetrazolium bromide (MTT)] using an automated microplate reader (Bio-rad, Hercules, CA, USA). Active compounds were then screened for OPC proliferation and differentiation assessing [3 H]thymidine incorporation and the expression of transcripts for the CGT and MBP markers of early and late stage of oligodendrocyte differentiation, respectively.

#### Score assignment

For the combined analysis of proliferation (Prolif), early differentiation (Diff_CGT) and late differentiation (Diff_MBP) data, we assigned a score to each of the experimental setting performed in purified OPC cultures: MTT score = 1, Prolif score = 10, Diff_CGT score = 3, Diff_MBP score = 5. Score numbers have been assigned with the aim of: (i) isolate MTT contribution [range 0–3]; (ii) keep Prolif scored contribution within the same range of Diff_ CGT and MBP [range 0–60]; (iii) reward both Diff_CGT and Diff_MBP more than proliferation since the final stage of oligodendrocyte developmental program is impaired during the remyelination process^25^; (iv) reward Diff_MBP more than Diff_CGT since MBP mRNA is subjected to a broader range of expression changes with respect to CGT mRNA during the *in vitro* developmental period analyzed [range 0–300] (Table 2).

### Mixed glial cell cultures

Cells enzymatically dissociated from forebrains of newborn CD1 Swiss mice (1.2 × 10^5^ cells/cm^2^) were grown in Dulbecco’s modified eagle medium containing 10% Foetal Bovine Serum (FBS), 2 mM glutamine and penicillin (50 μg/ml) and streptomycin (50 μg/ml); all media, sera and reagents were by GIBCO (Lifetechnologies, Grand Island, NY). After 9–10 DIV, when small groups of OPC started to proliferate on the top of the astroglia layer together with microglial cells, medium was replaced with a defined serum-free medium without thyroid hormones^56^. Cells were then incubated with the selected compounds (10 μM) or DMSO (0.001% vehicle) for 48 h. T3 (30 ng/ml; Sigma-Aldrich) and T4 (40 ng/ml; Sigma-Aldrich) were used as positive controls.

### Organotypic cerebellar slice cultures

For myelination studies, organotypic cerebellar slices were prepared from mouse cerebellum as previously described for rat^26^. Briefly, slices were prepared from cerebellum of postnatal day 7 CD1 Swiss mice and grown in culture medium with compounds at 20 μM concentration or DMSO (0.002% vehicle) for 5 DIV (replacing fresh medium after 1 DIV and every 2–3 days). A compound dose two times higher than that used in monolayer cell cultures was used due to the thickness of the slices.

For remyelination studies, cerebellar slices were prepared from mouse cerebellum as previously described for rat^27^. Briefly, slices were prepared from cerebellum of postnatal day 10 CD1 Swiss mice and grown in culture medium for 7 DIV (replacing fresh medium after 1 DIV and every 2–3 days). Demyelination was then induced by addition of 0.5 mg/ml lysolecithin (Sigma-Aldrich) to the medium for 16 h. Two hours after toxin removal, cultures were incubated with the compounds (20 μM) or DMSO (0.002% vehicle) for 4 days (for gene expression experiments) or 7 days (for immunohistochemistry). Progesterone (40 μM, Sigma-Aldrich) was used as positive control.

### Automated immunohistochemistry quantification of remyelination

We used a LSM 5 Pascal Laser Scanning Microscope to obtain stacks of photographs of MBP and NFH immunolabelling at 1 μm intervals in white matter areas at ×20 magnification in remyelinating slices with or without drugs. For each experimental point, 3 stacks were analyzed in three separate experiments in a blinded fashion by two different investigators. We developed an image analysis method to automatically quantify only MBP staining overlying axons. We implemented a statistical evaluator based on a sound and accepted methodology^57^ for pixel quantification which are both green and red above a defined intensity overlap, producing a mask of co-localization. The value of co-localization of each layer within a confocal stack was divided for the corresponding NFH staining to form the “Index of remyelination”. Our statistic combined three steps: (i) a split channel filter that isolates different color channels in a given image stack; (ii) a sub-color palette matching algorithm (e.g., this was used to sample the presence of the yellow color according to the palette); (iii) the assessment of biological expression co-localization^58^.

### Analysis of purity

HPLC quantitative determination of the purity of edaravone and 5-methyl-7-methoxyisoflavone was performed by Varian Pro star (waters symmetry C18 25 × 4.6 5 μm, loop 10 μl, 254 nm). High resolution mass spectrum was obtained with the spectrometer Mariner Applied Biosystems ESI-TOF. Spectra NMR (DMSO-300K) were registered using a spectrometer Bruker AVANCE III 400 MHz Ultrashield Plus.

### Chemical analog selection

Search in public Database (PubChem, Zinc and ChemSpider) of chemical analogs was performed with different approaches: bio-dimensional similarity using Tanimoto score calculated from 2D structure fingerprint and substructure search using main scaffolds.

### Chemical Similarity Network Analysis

We used Chemical Similarity Network Analysis Pull-down (CSNAP; http://services.mbi.ucla.edu/CSNAP; University of California, Los Angeles) as a computational method to look for a possible consensus target of the hit compounds identified through the phenotype-based screening. The technique behind CSNAP utilizes chemical similarity networks for chemotype (consensus chemical pattern) recognition and drug target profiling. The result of this technique is presented via a Schwikowski-score that associates a compound to a list of targets (Supplementary Dataset S2) through the generation of ligand-target interaction fingerprint^59^. The objective of this analysis was the identification of robust interaction networks^60^ and the identification of a possible consensus target, i.e. a target shared by the majority of the candidate compounds that previously showed an effect when analyzed singularly *in vitro* and *ex vivo*.

### Statistical analysis

For all the experiments in which statistical analysis was performed, a paired Student’s t-test was used. p ≤ 0.05 was considered significant.

## Additional Information

**How to cite this article:** Eleuteri, C. *et al*. A staged screening of registered drugs highlights remyelinating drug candidates for clinical trials. *Sci. Rep.* **7**, 45780; doi: 10.1038/srep45780 (2017).

**Publisher's note:** Springer Nature remains neutral with regard to jurisdictional claims in published maps and institutional affiliations.

## Supplementary Material

Supplementary Information

Supplementary Dataset S1

Supplementary Dataset S2

## Figures and Tables

**Table 1:** Compound effect on OPC metabolic activity.

Category	Compound	Average	SD
**VASODILATOR**	VINPOCETINE	1.36	0.66
	ISOXSUPRINE	1.54	0.23
	NYLIDRIN	1.60	0.00
**STEROIDS**	CYPROTERONE	1.29	0.38
	CHLORMADINONE ACETATE	1.69	0.30
	ESTRADIOL (2X)	1.30	0.17
	FLUDROCORTISONE ACETATE	1.35	0.05
**FLAVONOIDS**	BIOCHANIN A	2.13	0.29
	QUERCITRIN	1.40	0.29
	5-METHYL-7-METHOXYISOFLAVONE	1.53	0.23
**ANTIHYPERTENSIVE ACE INHIBITOR**	PERINDOPRIL ERBUMINE	1.30	0.21
	TRANDOLAPRIL	1.30	0.13
**ANTIHYPERTENSIVE SARTAN**	LOSARTAN	1.30	0.18
	OLMESARTAN	1.31	0.17
**ANTIHYPERTENSIVE DIURETIC**	DIAZOXIDE	1.32	0.05
	VERATRINE SULFATE	1.40	0.20
**ANTIHYPERTENSIVE β-BLOCKER**	ALPRENOLOL	1.37	0.25
**ANTIINFLAMMATORY**	HYDROXYTOLUIC ACID	1.33	0.06
	S-(1,2-DICARBOXYETHYL)GLUTATHIONE	1.32	0.21
	VULPINIC ACID	1.37	0.35
	DIACERIN	1.33	0.06
	FLUOCINOLONE ACETONIDE	1.40	0.30
	BECLOMETHASONE DIPROPIONATE	1.51	0.14
**ANTIBACTERIC**	FENAMISAL	1.33	0.21
	KANAMYCIN A SULPHATE	1.32	0.03
	GENTAMICIN SULPHATE	1.44	0.15
	HYGROMYCIN B	1.33	0.25
**ANTIHISTAMINIC**	TRIPELENNAMINE CITRATE	1.40	0.26
	DIPHENYLPYRALINE HYDROCHLORIDE	1.48	0.16
	ORPHENADRINE CITRATE	1.49	0.14
**ANTI-DEPRESSIVE**	ESCITALOPRAM OXALATE	1.43	0.12
**ANTIOXIDANT**	EDARAVONE	1.33	0.15
**MUSCARINIC ANTAGONIST**	HYOSCYAMINE	1.53	0.12
**HYPOLIPIDEMIC**	LOVASTATIN	1.23	0.10
	NIACIN	1.47	0.06
**CATHARTIC**	PHENOLPHTHALEIN	1.67	0.29
**COMT INHIBITOR**	3,5-DINITROCATECHOL (OR-486)	1.37	0.06
**MUSCARINIC AGONIST**	AMINOBENZTROPINE	1.33	0.06
**PSYCHOSTIMULANT**	URIDINE TRIPHOSPHATE TRISODIUM	1.40	0.10
**ANTI-INFECTIVE**	CINEOLE	1.38	0.35
**ANTIARYTHMIC**	LIDOCAINE HYDROCHLORIDE	1.55	0.21
**LIPASE INHIBITOR**	ORLISTAT	1.32	0.21
**ANTIFUNGAL**	AVOCADYNE ACETATE	1.43	0.12

All 2,000 compounds of the library were screened blind by MTT assay in purified OPC cultures; table shows compound names and pharmacological categories of the 42 more active compounds. MTT value was quantified by an Efficacy Ratio defined as: ER = absorbance of drug/absorbance of untreated control. The 42 drugs showing a mean value of ER ≥ 1.3 and suitable for human use in their present chemical form were selected. Lovastatin, a drug that presented a mean value of ER = 1.23 ± 0.1 was added to this list because of the demonstrated activity of this compound on OPC differentiation and myelin formation and used as internal control of the screening.

**Table 2:** Raw data of the activity of the 43 selected compounds originating Figure 2.

Compound	Prolif raw	Prolif SE	Prolif Score	Prolif Total	Diff-CGT raw	Diff-CGT SE	Diff_CGT Score	Diff_CGT Total	Diff_MBP raw	Diff_MBP SE	Diff_MBP Score	Diff_MBP Total	TOTAL SCORE
**PDGF**	3.17	0.98	10.00	**31.75**	**—**	**—**	**—**	**—**	**—**	**—**	**—**	**—**	**—**
**T3 + T4**	**—**	**—**	**—**	**—**	4.21	0.49	3.00	**12.63**	8.95	1.98	5.00	**44.75**	**—**
**VULPINIC ACID**	2.11	0.59	10.00	**21.08**	11.19	4.49	3.00	**33.58**	60.00	54.54	5.00	**300.00**	**354.65**
**LOVASTATIN**	2.94	0.26	10.00	**29.36**	22.86	20.35	3.00	**68.57**	58.25	55.69	5.00	**291.24**	**389.17**
**EDARAVONE**	3.52	0.64	10.00	**35.19**	23.08	16.09	3.00	**69.25**	48.11	42.19	5.00	**240.55**	**344.99**
**FENAMISAL**	3.15	0.41	10.00	**31.55**	9.52	5.06	3.00	**28.57**	36.89	29.81	5.00	**184.47**	**244.59**
**5-METHYL-7-METHOXYISOFLAVONE**	7.05	1.36	10.00	**70.54**	8.86	0.34	3.00	**26.59**	35.95	20.86	5.00	**179.73**	**276.86**
**LOSARTAN**	1.14	0.23	10.00	**11.44**	11.11	10.23	3.00	**33.34**	23.24	21.14	5.00	**116.21**	**160.98**
**CHLORMADINONE ACETATE**	0.66	0.24	10.00	**6.61**	10.09	0.60	3.00	**30.26**	16.79	1.26	5.00	**83.96**	**120.83**
**BIOCHANIN A**	1.24	0.08	10.00	**12.38**	3.53	3.39	3.00	**10.58**	7.96	7.15	5.00	**39.79**	**62.75**
**CYPROTERONE**	1.25	0.34	10.00	**12.49**	2.46	0.13	3.00	**7.39**	7.65	0.06	5.00	**38.23**	**58.12**
**OLMESARTAN**	0.79	0.17	10.00	**7.88**	4.06	3.08	3.00	**12.19**	6.49	4.47	5.00	**32.44**	**52.50**
**FLUOCINOLONE ACETONIDE**	0.59	0.17	10.00	**5.89**	2.40	0.13	3.00	**7.21**	5.95	0.48	5.00	**29.74**	**42.83**
**TRANDOLAPRIL**	2.84	1.24	10.00	**28.35**	6.06	5.44	3.00	**18.17**	5.86	5.11	5.00	**29.31**	**75.83**
**QUERCITRIN**	3.90	0.44	10.00	**38.96**	4.68	1.76	3.00	**14.05**	5.10	3.09	5.00	**25.48**	**78.48**
**URIDINE TRIPHOSPHATE TRISODIUM**	4.80	0.36	10.00	**47.99**	3.75	0.21	3.00	**11.26**	5.02	0.41	5.00	**25.10**	**84.35**
**DIPHENYLPYRALINE HYDROCHLORIDE**	3.65	1.35	10.00	**36.46**	3.10	2.01	3.00	**9.29**	4.90	3.23	5.00	**24.52**	**70.27**
**VERATRINE SULFATE**	3.23	0.81	10.00	**32.33**	4.09	0.17	3.00	**12.27**	4.82	0.27	5.00	**24.11**	**68.72**
**3,5-DINITROCATECHOL**	5.55	1.35	10.00	**55.47**	4.74	0.39	3.00	**14.22**	4.76	0.19	5.00	**23.78**	**93.48**
**HYGROMYCIN B**	3.66	0.45	10.00	**36.65**	4.46	4.08	3.00	**13.37**	4.65	3.69	5.00	**23.25**	**73.27**
**ESCITALOPRAM OXALATE**	3.86	0.56	10.00	**38.63**	3.91	3.31	3.00	**11.72**	3.89	1.53	5.00	**19.46**	**69.80**
**DIAZOXIDE**	1.34	0.11	10.00	**13.37**	3.31	2.32	3.00	**9.92**	3.67	1.25	5.00	**18.35**	**41.65**
**ESTRADIOL**	1.85	0.61	10.00	**18.47**	1.53	0.34	3.00	**4.59**	2.75	1.10	5.00	**13.76**	**36.83**
**AMINOBENZTROPINE**	5.77	1.17	10.00	**57.67**	1.48	*	3.00	**4.44**	2.58	*	5.00	**12.92**	**75.04**
**DIACERIN**	1.28	0.36	10.00	**12.76**	0.19	*	3.00	**0.58**	2.56	*	5.00	**12.81**	**26.16**
**PERINDOPRIL ERBUMINE**	2.33	0.32	10.00	**23.28**	1.76	0.11	3.00	**5.27**	2.45	0.10	5.00	**12.23**	**40.78**
**PHENOLPHTHALEIN**	1.21	0.10	10.00	**12.11**	2.85	1.68	3.00	**8.55**	2.44	1.53	5.00	**12.19**	**32.85**
**ORPHENADRINE CITRATE**	1.08	0.39	10.00	**10.84**	0.41	*	3.00	**1.24**	1.66	*	5.00	**8.29**	**20.36**
**BECLOMETHASONE DIPROPIONATE**	0.48	0.07	10.00	**4.78**	1.06	*	3.00	**3.17**	1.55	*	5.00	**7.74**	**15.69**
**NIACIN**	0.77	0.21	10.00	**7.75**	0.70	*	3.00	**2.11**	1.35	*	5.00	**6.73**	**16.58**
**FLUDROCORTISONE ACETATE**	0.74	0.13	10.00	**7.41**	0.76	*	3.00	**2.28**	1.28	*	5.00	**6.41**	**16.10**
**AVOCADYNE ACETATE**	1.09	0.15	10.00	**10.88**	1.20	*	3.00	**3.61**	1.10	*	5.00	**5.48**	**19.97**
**KANAMYCIN A SULPHATE**	0.95	0.20	10.00	**9.45**	1.50	*	3.00	**4.51**	1.03	*	5.00	**5.15**	**19.11**
**LIDOCAINE HYDROCHLORIDE**	2.43	0.14	10.00	**24.29**	1.10	*	3.00	**3.30**	1.02	*	5.00	**5.09**	**32.67**
**S-(1,2-DICARBOXYETHYL)GLUTATHIONE**	1.37	0.15	10.00	**13.66**	0.71	*	3.00	**2.12**	0.85	*	5.00	**4.23**	**20.01**
**HYOSCYAMINE**	1.35	0.40	10.00	**13.52**	0.43	*	3.00	**1.30**	0.84	*	5.00	**4.18**	**19.00**
**VINPOCETINE**	0.83	0.26	10.00	**8.31**	0.96	*	3.00	**2.89**	0.62	*	5.00	**3.11**	**14.31**
**GENTAMICIN SULPHATE**	1.16	0.21	10.00	**11.59**	0.67	*	3.00	**2.02**	0.54	*	5.00	**2.69**	**16.30**
**ORLISTAT**	1.87	0.36	10.00	**18.66**	0.70	*	3.00	**2.09**	0.53	*	5.00	**2.65**	**23.41**
**ISOXSUPRINE**	1.20	0.40	10.00	**12.00**	0.95	*	3.00	**2.84**	0.49	*	5.00	**2.46**	**17.31**
**CINEOLE**	1.03	0.30	10.00	**10.26**	0.63	*	3.00	**1.90**	0.45	*	5.00	**2.24**	**14.40**
**TRIPELENNAMINE CITRATE**	3.61	0.92	10.00	**36.08**	0.21	*	3.00	**0.63**	0.22	*	5.00	**1.10**	**37.81**
**NYLIDRIN**	0.94	0.07	10.00	**9.38**	0.09	*	3.00	**0.27**	0.21	*	5.00	**1.04**	**10.68**
**ALPRENOLOL**	3.57	0.33	10.00	**35.69**	0.23	*	3.00	**0.70**	0.09	*	5.00	**0.43**	**36.83**
**HYDROXYTOLUIC ACID**	2.26	0.29	10.00	**22.57**	0.22	*	3.00	**0.65**	0.05	*	5.00	**0.24**	**23.45**

We assigned a different score to each experimental setting performed in purified OPC cultures, rewarding more late differentiation (Diff_MBP) than early differentiation (Diff_CGT) and both more than proliferation (Prolif; see “score assignment” in method section for details). The score contribution of each compound was derived by multiplying the assigned score to the raw data of the experimental setting it belongs to. Score contributions have been then summed per-compound (Total score). The asterisk indicates the drugs that have not achieved the established thresholds in the first gene expression analysis and were not tested again. The following positive controls were used: PDGF (20 ng/ml) for proliferation and T3 (30 ng/ml) and T4 (40 ng/ml) for differentiation assays.

**Table 3:** Compound effect on OPC differentiation in cultures of mixed glial cells.

Compound	MBP RNA level (Mean ± SE)	p-value	CGT RNA level (Mean ± SE)	p-value
CTR	1 ± 0	—	1 ± 0	—
T3 + T4	2.85 ± 0.45	**0.01**	2.65 ± 0.9	0.09
VULPINIC ACID	1.72 ± 0.49	0.1075	1.34 ± 0.47	0.2549
LOVASTATIN	2.84 ± 0.53	**0.0127**	1.78 ± 0.18	**0.006**
METHOXYISOFLAVONE	2.32 ± 0.5	**0.039**	1.97 ± 0.34	**0.051**
EDARAVONE	3.86 ± 0.39	**0.0009**	2.76 ± 0.58	**0.0197**
CHLORMADINONE ACETATE	3.12 ± 0.57	**0.0101**	2.35 ± 0.56	**0.037**
LOSARTAN	2.38 ± 0.63	**0.0467**	2.8 ± 0.57	**0.0532**
FENAMISAL	2.78 ± 0.76	0.0726	1.38 ± 0.22	0.1106

The 7 selected compounds were screened for their ability to stimulate OPC differentiation in cultures of mixed glial cells. Cells were treated with compounds (10 μM) or DMSO (0.001% vehicle) for 48 h and the expression of CGT and MBP mRNA was evaluated by real time RT-PCR. The results show that 5 out of 7 compounds significantly (p < 0.05) stimulated the expression of both MBP and CGT genes compared to untreated control. 5-methyl-7-methoxyisoflavone is abbreviated as methoxyisoflavone. Data are expressed as 2^−ΔΔCt^ value relative to untreated control, using GAPDH as reference gene. T3 (30 ng/ml) and T4 (40 ng/ml) were used as positive control.
