# Supplementary material for: A staged screening of registered drugs highlights remyelinating drug candidates for clinical trials
Source: Sci Rep. 2017 Apr 7;7:45780. doi: 10.1038/srep45780 (PMC5384285; doi:10.1038/srep45780)
Supplement: Supplementary Information [file srep45780-s1.pdf]

A staged screening of registered drugs highlights remyelinating drug candidates for clinical trials

C. Eleuteri [1, +], S. Olla [2, +], C. Veroni [1, +], R. Umeton [3], R. Mechelli[3], S. Romano[3], M.C. Buscarinu[3], F. Ferrari [4], G. Calò [4], G. Ristori [3,*] , M. Salvetti [3,5 +,*] and C. Agresti [1, +,*]

[1] *Department of Neuroscience, Istituto Superiore di Sanità, 00161 Rome, Italy*

[2] *Istituto di Ricerca Genetica e Biomedica, Consiglio Nazionale delle Ricerche, Monserrato 09042, Italy*

[3] *Center for Experimental Neurological Therapies, Sant'Andrea Hospital, Department of Neurosciences, Mental Health and Sensory Organs (NESMOS), Faculty of Medicine and Psychology, Sapienza University of Rome, Italy*

[4] *Department of Medical Science, Section of Pharmacology and National Institute of Neuroscience, University of Ferrara, 44121 Ferrara, Italy*

[5] *IRCCS Istituto Neurologico Mediterraneo (INM) Neuromed, 86077 Pozzilli, IS, Italy*

*marco.salvetti@uniroma1.it; cristina.agresti@iss.it; giovanni.ristori@uniroma1.it

+ These authors contributed equally to this work

SUPPLEMENTARY METHODS

Purified OPC cultures

OPC were obtained from neonatal mouse primary mixed glial cultures. In brief, forebrains of newborn CD1 Swiss mice were freed of meninges, chopped into 0.2-mm sections and dissociated by mild trypsinization procedure and gentle mechanical disruption with a Pasteur pipette. The cells were seeded into poly-L-lysine (10 µg/ml, Sigma-Aldrich, St. Louis, MO) coated 60 mm diameter culture dishes at the density of 1.2×10^5 cells/cm2 and grown at 37°C in a 91.5% air–8.5% CO_2 humidified atmosphere in Dulbecco's modified eagle medium (DMEM) containing 10% Foetal Bovine Serum (FBS), 2 mM glutamine and penicillin (50 µg /ml) and streptomycin (50 µg/ml), replacing fresh medium after 1 DIV and every 2-3 days (media, sera and reagents by GIBCO, Lifetechnologies, Grand Island, NY). After 8-10 days, OPC were detached from astroglia layer by mechanical dissociation; to minimize contamination by microglial cells, the detached cell suspension was incubated for 1 hour at 37°C in a 175 cm2 culture flask. The non-adering cells were seeded in the same medium as above at the density of 1 × 105 cells/cm2 into poly-L-lysine-coated dishes. Two hours after plating, culture medium was replaced with defined serum-free DMEM without thyroid hormones (56). Macrophage/microglia contamination accounted for less than 1% of total cells, as assessed by immunostaining with the monoclonal antibody (mAb) cd11b (AbD Serotec, Oxford, UK); glial fibrillary acid protein-positive astrocytes were virtually absent and the majority of cells (>99%) belonged to the OL lineage, as assessed by specific mAbs, which bind to differentiation-regulated surface antigens of these cells.

MTT reduction

One day after plating, purified OPC subcultured in 96-well microtiter plates were incubated with or without drugs (10 µM) in DMSO (0.001% vehicle) for 48 h. Measurement of reduction of [3-(4,5-dimethylthiazol-2-yl)-2,5-diphenyltetrazolium bromide (MTT)] was carried out as previously described (56). MTT (Sigma-Aldrich) was added to the culture medium at final concentration of

0.25 mg/ml during the final 4 h of incubation. Platelet derived growth factor (PDGF,20 ng/ml, Preprotech, Rocky Hill, NJ, USA) and tri-iodothyronin (T3, 30 ng/ml) and thyroxine (T4, 40 ng/ml) (both from Sigma-Aldrich), known to stimulate oligodendrocyte proliferation and differentiation, respectively, were used as positive controls.

[3H] Thymidine incorporation assay

Purified OPC subcultured in 96-well microtiter plates were exposed after 1 day to the compounds (10 µM) or to DMSO alone (0.001% vehicle) for 48 h. [3H]Thymidine (5 mCi/mL; [methyl 3H]thymidine, 70–85 Ci/mmol; Amersham, Bucks., UK) was added during the final 4 h of the experiment. The cells were harvested using a 96 well plate automated cell harvester and the radioactivity was measured by a microplate scintillation counter (Top-Count, Packard Instrument Co., Meriden, CT, USA). PDGF (20 ng/ml), was used as positive control.

Quantitative real-time RT-PCR assay

OPC, mixed glial cells and cerebellar slices were incubated in the absence or presence of compounds or specific positive controls for the indicated times. Total RNA was extracted using RNeasy Mini kit (QIAGEN, Valencia, CA), according to the manufacturer's instructions. For each sample, one microgram of RNA, measured by Nanodrop (Thermo Fisher Scientific, Waltham, MA, USA), was reverse transcribed using High Capacity Reverse Transcription kit (Lifetechnologies) and then analyzed by real-time PCR, using the ABI PRISM 7500 Real Time PCR, Gene Expression Master Mix and inventoried gene expression assays (all from Lifetechnologies). The relative amounts of ceramide-galactosyltransferase (CGT; assay code: Mm00495930_m1) and myelin basic protein (MBP; assay code: Mm01266402_m1) transcripts were calculated using the comparative Ct method and normalized to the internal GAPDH control (GAPDH For: ACC CAC CCC AGC AAG GA; GAPDH Rev: GAA ATT GTG AGG GAG ATG CTC AGT; GAPDH Probe: VIC-AAG AGA GGC CCT ATC C-MGB).

Immunohistochemistry

Cerebellar slices were incubated in the absence or presence of compounds, fixed in 4% paraformaldehyde in PBS for 10 min and then stained using double indirect immunofluorescence techniques. After washing with PBS and a blockade with 10% normal donkey serum (Sigma-Aldrich) in PBS for 1 h, sections were incubated overnight at 4°C with anti- neurofilament heavy chain (NFH) (pAb (1:500, AbD Serotec) and anti-MBP mAb (clone smi99, 1:1000, Covance, Denver, PA) diluted in PBS containing 1% BSA and 0.25% Triton. Bindings were visualized using a mixture of CY3-conjugated donkey anti-rabbit IgG and fluorescein-conjugated donkey anti-mouse IgG (Jackson ImmunoResearch Laboratories, West Grove, PA) diluted in PBS+1% normal serum. The incubation with the secondary Abs was carried out for 1 h at room temperature. Sections were then mounted in Vectashield (Vector Laboratories, Burlingame, CA) and sealed. Negative controls were performed by replacing primary antibodies with preimmune serum or IgG isotype control.

Calcium mobilization assay

HEK293mNPSR were generated as previously described (30). Cells were cultured in culture medium consisting of DMEM +10%FBS, penicillin (100 IU/ml), streptomycin (100 µg/ml), L-glutammine (2 mM), fungizone (1 µg/ml) and hygromycin B (100 µg/ml) and treated as previously described (30). After placing cell culture and compound plates into the FlexStation II (Molecular Devices, Sunnyvale, CA, USA), fluorescence changes were measured after 10 min of stabilization at 37°C (30). In antagonism experiments, SHA 68 and compounds were injected into the wells 24 min before adding NPS. All cell culture media and supplements were from Invitrogen (ThermoFisher Scientific). All other reagents were from Sigma Chemical Co. (Poole, U.K.) and were of the highest purity available. NPS and SHA 68 were synthesized in the Department of Chemical and Pharmaceutical Sciences (University of Ferrara, Italy) by Prof. Guerrini and Prof. Trapella, respectively. NPS was solubilized in bidistilled water at a final concentration of 1 mM, while SHA 68 and all compounds were in DMSO at a final concentration of 10 mM. Stock solutions of ligands were stored at -20 °C.

Terminology and data analysis of calcium mobilization experiments

The pharmacological terminology adopted in calcium mobilization experiments is consistent with IUPHAR recommendations. For potency values 95% confidence limits were indicated. Maximum change in fluorescence, expressed as percent over the baseline fluorescence, was used to determine agonist response. Agonist potencies are given as pEC50 i.e. the negative logarithm to base 10 of the molar concentration of an agonist that produces 50% of the maximal effect of that agonist. Concentration-response curves to agonists were fitted to the classical four-parameter logistic nonlinear regression model:

$$\text{Effec t} = \text{Baseline} + \frac{(E_{max} - \text{Baseline})}{(1 + 10^{(LogEC_{50} - Log_{[compound]})Hillslope})}$$

EC50 is the concentration of agonist producing a 50% maximal response and n is the Hill coefficient of the concentration-response curve to the agonist. Curve fitting was performed using PRISM 6.0 (GraphPad Software In., San Diego, USA).

Antagonist potencies were derived from Gaddum Schild equation:

$$pA_2 = -\log\left[\frac{\text{CR } -1}{\text{antagonist}}\right]$$

assuming a slope value equal to unity, where CR indicates the ratio between agonist potency in the presence and absence of antagonist.

SUPPLEMENTARY FIGURES

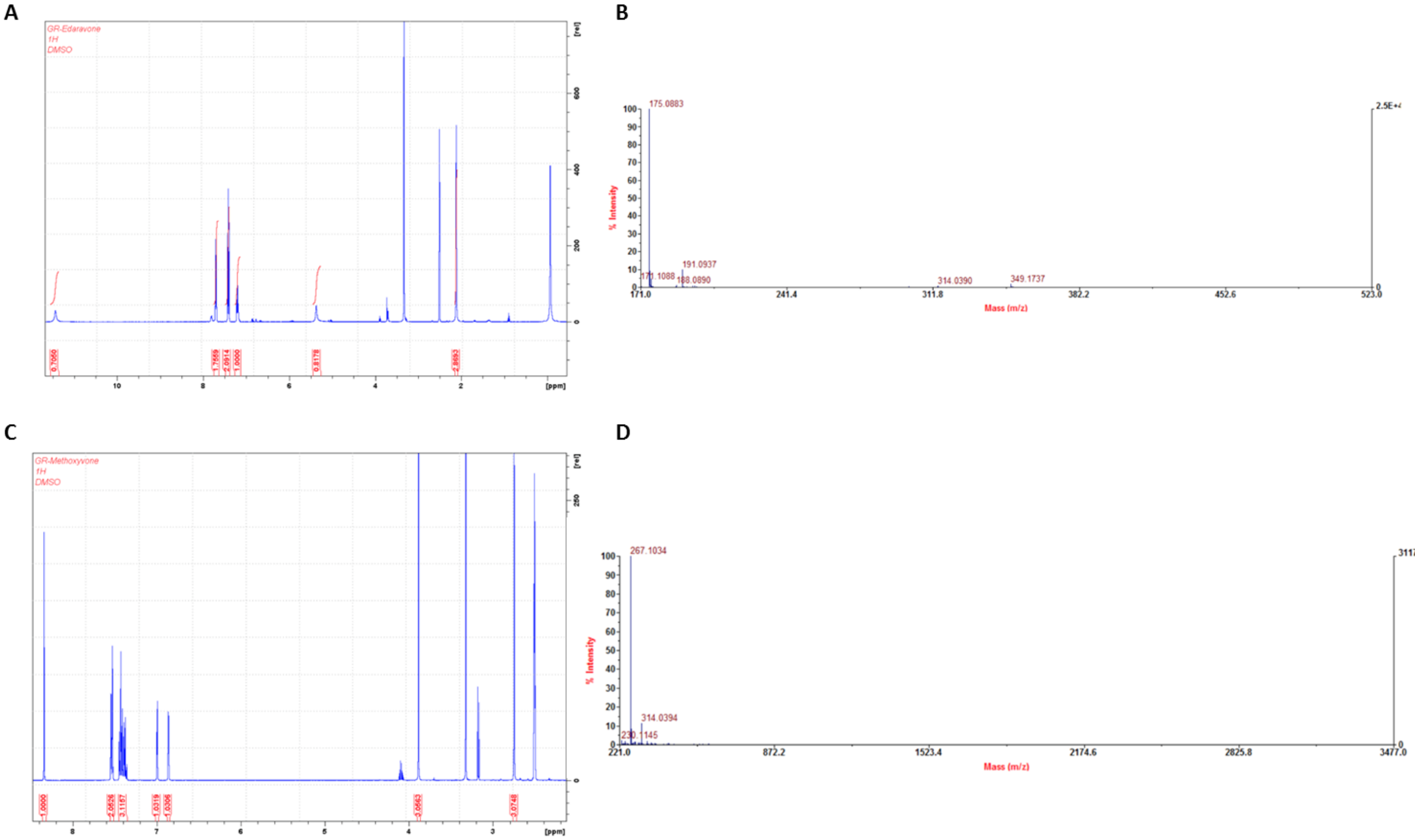

Supplementary Figure S1- Analysis of purity of the two hit compounds - The analytical characterization of purity of the 2 top compounds was performed by Proton Nuclear Magnetic Resonance Spectroscopy (A and C) and Liquid chromatography–mass spectrometry (B and D) techniques. Results confirmed that the chemical compounds analyzed correspond to edaravone with a purity of 97% (A-B) and 5-methyl-7-methoxyisoflavone with a purity of 99%, (C-D), showing that their activity depends exclusively to compounds.

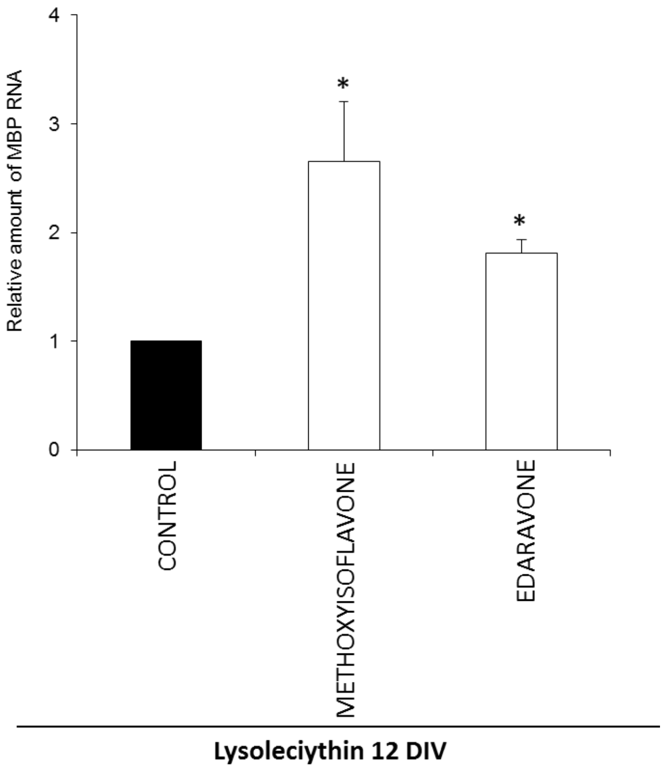

Supplementary Figure S2- Hit compound retesting. Slices (from P10 mouse cerebellum) were treated with lysolecithin for 16 hour and then incubated with new stocks of the two hits (20µM) immediately after toxin removal. After 4 DIV total RNA was extracted and reverse-transcribed and the expression of MBP transcript was evaluated by real time RT-PCR. Results show that repurchased batches of both edaravone and 5-methyl-7-methoxyisoflavone (methoxyisoflavone) significantly induced MBP RNA compared to control slices confirming the remyelinating potential of the two hits and avoiding risk of false positives. (*$p \leq 0.05$).

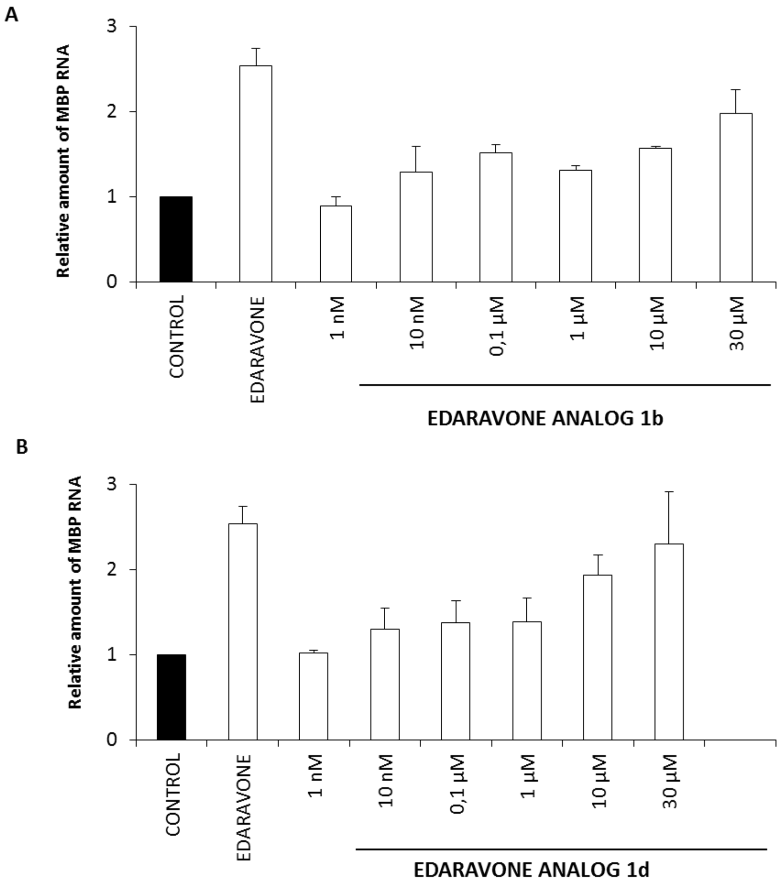

Supplementary Figure S3 - Dose-response curves of edaravone analogs. Cells from mixed glial cultures were incubated with edaravone active analogs 1b (A) and 1d (B) at 6 different concentrations (0.001 – 30 µM) for 48h. Expression of MBP mRNA was evaluated by real time RT-PCR. The results shown that both analogs dose-dependently stimulated MBP RNA. Values represent the mean ± SEM of 3 independent experiments.

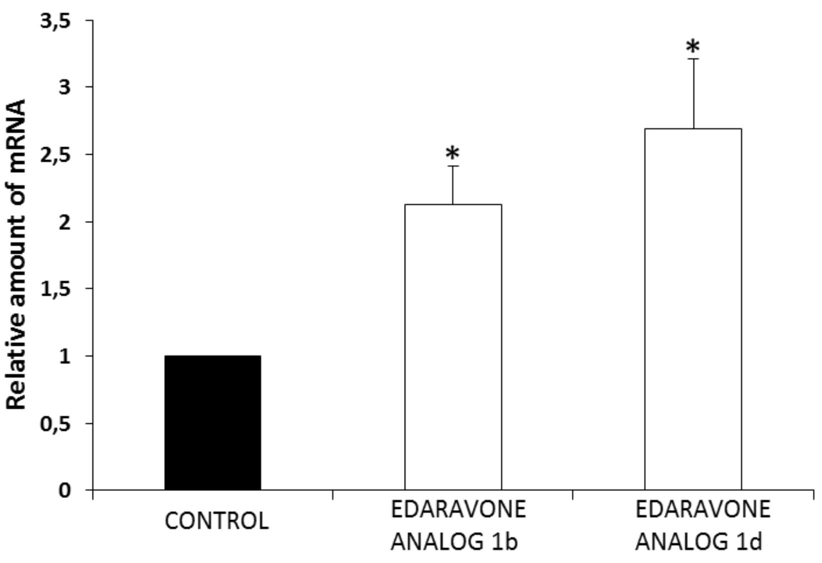

Supplementary Figure S4 – Effect of chemical analogues of edaravone on MBP RNA expression. Two out of four structural analogues of edaravone, selected by computational methods, were able to induce MBP transcript in lysolecithin-demyelinated cerebellar slices. These results confirmed and validated the chemical structure and biological activity of the compound. Values represent the mean ± SEM of 3 independent experiments. (*p≤0.05).

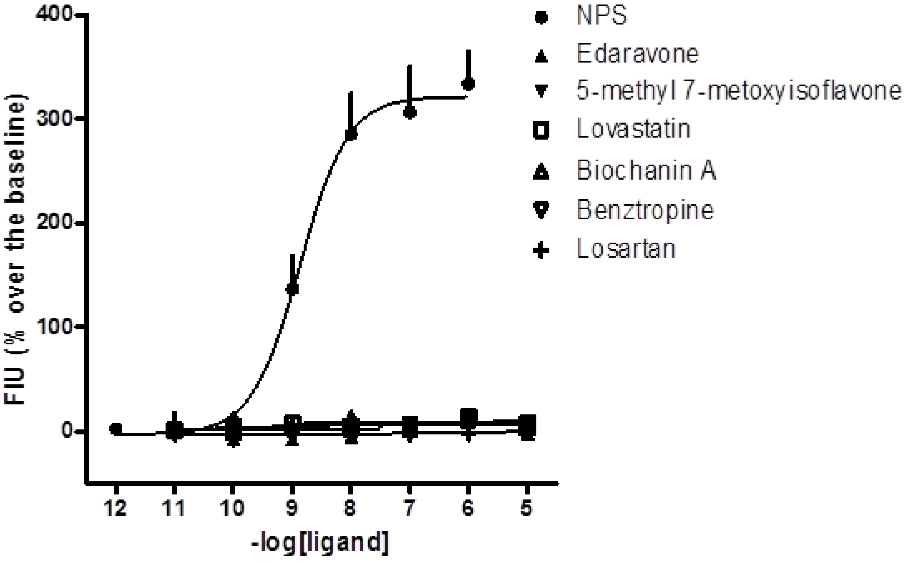

Supplementary Figure S5. Analysis of the agonistic activity of the compounds on NPSR. Calcium mobilization assay was performed in HEK293mNPSR cells to test the dose response activity of NPS, edaravone, 5-methyl 7-metoxyisoflavone, lovastatin, biochanin A, benztropine and losartan. The compounds did not stimulate calcium mobilization. Data are expressed as mean ± SEM of 3 separate experiments run in duplicate.

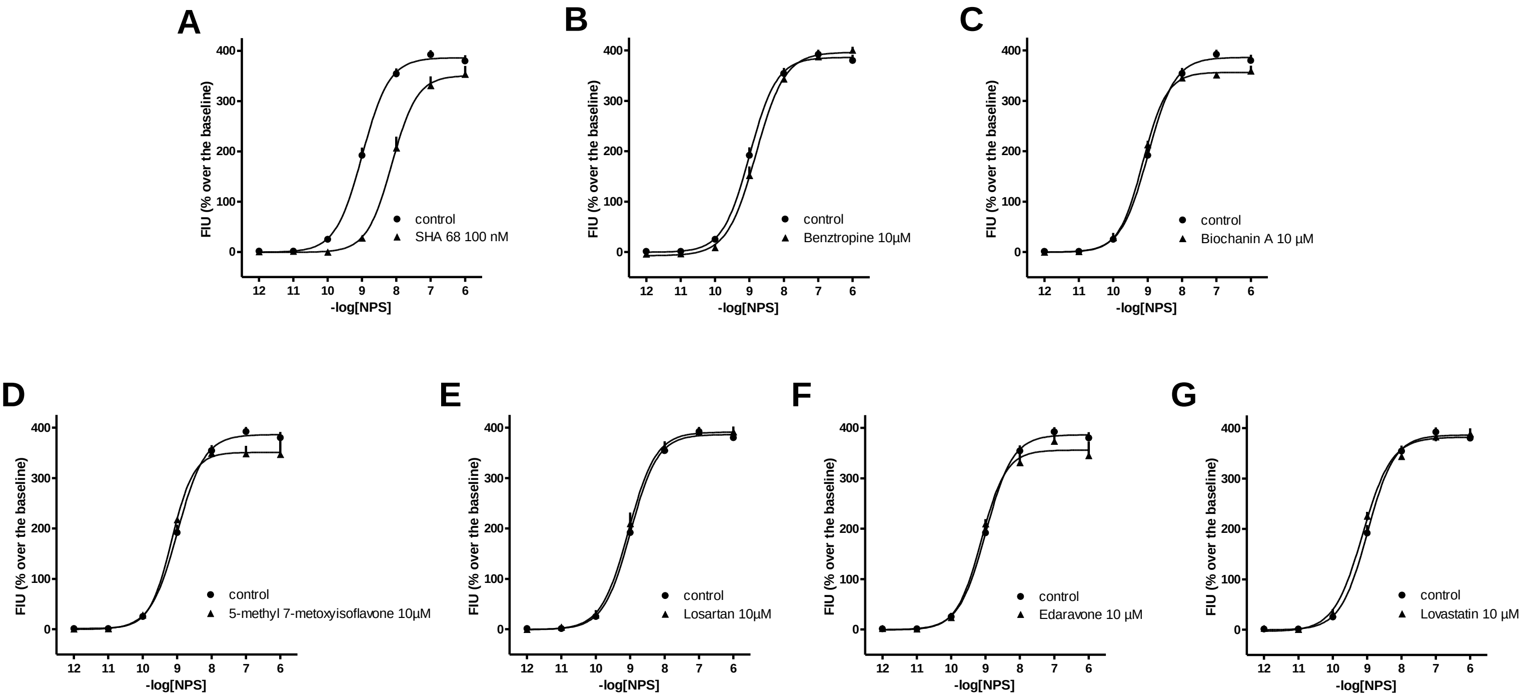

Supplementary Figure S6. Analysis of the antagonistic activity of the compounds on NPSR . Calcium mobilization assay was performed in HEK293mNPSR cells to test the dose response activity to NPS in absence (control) and in presence of 100 nM SHA 68 (panel A) and 10 µM of benztropine (panel B), biochanin A (panel C), 5-methyl 7-metoxyisoflavone (panel D), losartan (panel E), edaravone (panel F) and lovastatin (panel G). Compounds did not affect the concentration response curve to NPS. Data are expressed as mean ± SEM of 3 separate experiments run in duplicate.
